# Clinical characteristics and risk factors in patients with colon cancer liver metastases

**DOI:** 10.3389/fonc.2026.1817217

**Published:** 2026-07-22

**Authors:** Shan Ou, Qian Li, Peng Shi

**Affiliations:** Department of Gastroenterology, The First Affiliated Hospital of Chongqing Medical and Pharmaceutical College, Chongqing, China

**Keywords:** carcinoembryonic antigen, colon cancer, liver metastasis, logistic regression, lymphovascular invasion, perineural invasion, risk factors

## Abstract

**Background:**

Liver metastases (LM) are clinically important in colon cancer, but routinely available clinicopathological and laboratory profiles associated with LM status remain incompletely characterized. This study aimed to identify clinical, pathological, and laboratory variables associated with LM status in patients with colon cancer.

**Methods:**

This retrospective observational study included patients with pathologically confirmed colon cancer diagnosed between January 2020 and December 2023. LM was identified by imaging and/or histopathological confirmation. A pragmatic 1:2 non-propensity-score matching strategy was used to select non-LM controls. Univariable and multivariable logistic regression analyses were performed to evaluate variables associated with LM status. Sensitivity analyses and exploratory subgroup analyses of synchronous and metachronous LM were conducted.

**Results:**

Among 756 eligible patients, 101 developed LM, yielding an incidence of 13.4%. The matched cohort included 101 LM patients and 202 controls. After adjustment, lymphovascular invasion (adjusted odds ratio [aOR] 2.57, 95% confidence interval [CI] 1.38–4.76; P = 0.003), perineural invasion (aOR 1.94, 95% CI 1.05–3.59; P = 0.035), N2 stage (aOR 2.48, 95% CI 1.17–5.25; P = 0.018), carcinoembryonic antigen (CEA) per doubling (aOR 1.72, 95% CI 1.42–2.10; P<0.001), and neutrophil-to-lymphocyte ratio (NLR) per 1-unit increase (aOR 1.35, 95% CI 1.15–1.59; P<0.001) were independently associated with LM status. Sensitivity analyses showed generally consistent findings. Synchronous LM was characterized by higher carcinoembryonic antigen and NLR levels than metachronous LM.

**Conclusions:**

LM status in colon cancer was associated with adverse pathological features, nodal burden, higher CEA levels, and higher NLR. These routinely available variables may serve as supportive clinical indicators for assessing hepatic involvement, but should not be interpreted as causal factors or as a validated prediction model.

## 1 Introduction

Colon cancer remains a leading contributor to cancer morbidity and mortality worldwide, and hepatic dissemination represents the most clinically consequential pattern of distant spread because it frequently determines resectability, systemic treatment selection, and long-term survival (1, 2). Contemporary multidisciplinary management has expanded the therapeutic landscape for colorectal cancer liver metastases (CRLM), including optimized perioperative systemic therapy, parenchymal-sparing liver surgery, ablative and transarterial techniques, and more refined response-adapted sequencing strategies (3, 4). However, population-level outcomes remain heterogeneous, and a substantial proportion of patients still present with synchronous liver metastasis or develop metachronous disease during surveillance, underscoring the need for improved risk stratification at diagnosis and during early treatment pathways (5, 6). Current clinical decision-making relies on an integrated assessment of radiologic staging, pathological risk features, and blood-based markers. International and regional guidelines emphasize high-quality baseline staging, standardized follow-up, and timely multidisciplinary evaluation for potentially liver-directed treatment, particularly in patients with potentially resectable or oligometastatic patterns (7). Nevertheless, guideline-recommended pathways do not fully resolve the practical problem of identifying, among patients with newly diagnosed colon cancer, those at highest risk of LM who may benefit from intensified hepatic assessment, closer imaging surveillance, and earlier referral to hepatobiliary teams (8).

Pathological invasion phenotypes and nodal tumor burden are biologically plausible correlates of metastatic potential. Features such as lymphovascular and perineural invasion (PNI) reflect enhanced capacity for intravasation, dissemination, and microenvironmental remodeling, while advanced nodal stage captures both tumor biology and regional disease extent. In parallel, blood-based biomarkers may provide complementary information (9, 10). Carcinoembryonic antigen (CEA) remains widely used for monitoring and surveillance despite imperfect sensitivity and specificity, and increasing attention has focused on systemic inflammation indices, including the neutrophil-to-lymphocyte ratio (NLR), as accessible surrogates of tumor–host immune interactions (11, 12). Although previous studies have identified individual clinicopathologic, serum-based, inflammatory, and molecular factors related to colorectal cancer liver metastasis, the integrated baseline profile of colon cancer patients with concurrent or subsequent liver metastases (LM) remains incompletely characterized in matched clinical cohorts (13–15). In particular, routinely available variables that can be obtained at initial diagnosis may have practical value for baseline staging and surveillance planning, but their associations with liver metastasis status and with synchronous versus metachronous presentation require further clarification (16, 17).

Therefore, we performed a retrospective matched cohort study to characterize clinical, pathological, and laboratory correlates of liver metastasis in colon cancer, to evaluate independent associations in multivariable models, and to explore differences between synchronous and metachronous LM within a clinically relevant time-based framework.

## 2 Methods

### 2.1 Study design

This retrospective observational study consecutively enrolled patients with pathologically confirmed colon cancer who were diagnosed and treated at our institution between January 2020 and December 2023. Eligible participants were adults with a definite diagnosis of primary colon adenocarcinoma based on surgical specimens or endoscopic biopsy, availability of complete baseline clinicopathologic data, and adequate imaging evaluation for metastatic assessment, including contrast-enhanced abdominal computed tomography (CT) and/or magnetic resonance imaging (MRI), with additional modalities when clinically indicated. Patients were excluded if they had rectal cancer or appendiceal malignancies, a prior or synchronous malignant tumor, LM originating from non-colonic primary lesions, incomplete key exposure/outcome data precluding risk-factor analysis, lack of evaluable pre-treatment imaging for liver staging, or receipt of anti-tumor therapy before definitive diagnostic assessment that could confound baseline measurements. Patients were categorized into a LM group if liver metastatic disease was identified at diagnosis or during follow-up based on imaging findings consistent with metastatic lesions and/or histopathological confirmation when available; those without evidence of LM throughout the observation period constituted the control group. Informed consent was obtained from all participants and/or their legal guardians. The study was approved by the institutional ethics committee and conducted in accordance with the Declaration of Helsinki and relevant guidelines. All data were anonymized prior to analysis to ensure confidentiality and protect participant privacy.

### 2.2 Grouping and outcome definitions

The primary outcome of this study was the occurrence of LM. Patients were grouped according to LM status during the study period. LM was ascertained using routine staging and follow-up assessments, primarily based on radiologic evidence and supported by clinical multidisciplinary evaluation. Specifically, LM was considered present when contrast-enhanced abdominal CT and/or MRI demonstrated hepatic lesions with imaging features consistent with metastatic disease, as documented in the formal radiology report and deemed compatible with colon cancer metastasis after multidisciplinary team (MDT) discussion or consensus clinical review. When imaging findings were equivocal or when histologic confirmation was clinically pursued, LM was confirmed by pathological evidence from liver biopsy or surgical resection specimens. The date of LM onset was defined as the earliest date on which the diagnostic criteria were first met, using the imaging examination date or the pathology report date, as appropriate. LM was further classified according to its temporal relationship with the initial diagnosis of colon cancer. Synchronous LM were defined as LM detected at the time of initial diagnosis or within 6 months thereafter, whereas metachronous LM were defined as LM first detected more than 6 months after the initial diagnosis during follow-up.

Accordingly, the LM group comprised patients with synchronous or metachronous LM identified at baseline or during follow-up. The control group comprised patients without evidence of LM throughout the entire observation period, as verified by all available follow-up imaging studies and clinical records. To minimize outcome misclassification, controls were required to have evaluable hepatic imaging and/or documented follow-up sufficient to exclude LM during the study window.

### 2.3 Data sources and data collection

Data were obtained from institutional information systems, including the electronic medical record (EMR), pathology database, radiology/imaging archive and reporting system, laboratory information system, operative and anesthesia records, and the structured follow-up registry. A standardized case report form was developed *a priori* to extract demographic information, clinicopathologic characteristics, laboratory indices, imaging findings, treatment-related variables, and outcome data. Data abstraction was independently performed by two trained investigators who were blinded to each other’s extraction results. After independent extraction, the two datasets were cross-checked for consistency. Any discrepancies were resolved through source-document verification, and disagreements that could not be reconciled were adjudicated by a third senior reviewer to ensure data accuracy and reproducibility. Missing data were prospectively documented during extraction. For each variable, the proportion and pattern of missingness were summarized. Missingness was categorized as critical (key variables required for exposure definition, outcome ascertainment, or prespecified multivariable modeling) versus noncritical (auxiliary covariates not essential for primary analyses). The handling strategy for missing data was prespecified, including complete-case analysis and, when appropriate, multiple imputation under an assumed missing-at-random mechanism.

### 2.4 Variables and measurements

Variables were predefined and collected at baseline, defined as the earliest assessment at the time of colon cancer diagnosis before initiation of definitive antitumor treatment. Demographic and clinical variables included age, sex, body weight, height, and body mass index (BMI, kg/m²), as well as smoking history, alcohol use, and comorbidities (including diabetes mellitus and hypertension); the American Society of Anesthesiologists (ASA) physical status classification was recorded for patients undergoing surgery. Tumor-related characteristics were extracted from endoscopic, operative, radiologic, and pathology reports, including primary tumor location (right colon, transverse colon, left colon, based on anatomical definitions), histologic subtype, differentiation grade, maximum tumor diameter, T stage, N stage, and overall TNM stage. Lymphovascular invasion (LVI), PNI, tumor deposits, and resection margin status were coded according to pathology documentation. The total number of examined lymph nodes and the number of positive lymph nodes were recorded, and the lymph node ratio (LNR) was calculated as positive nodes divided by examined nodes. Laboratory indices included baseline CEA and carbohydrate antigen 19-9 (CA19-9), complete blood count parameters (neutrophils, lymphocytes, platelets), and derived inflammatory indices (NLR = neutrophils/lymphocytes; PLR = platelets/lymphocytes). Liver function and nutrition-related biomarkers included alanine aminotransferase, aspartate aminotransferase, alkaline phosphatase, gamma-glutamyl transferase, total bilirubin, and albumin. Treatment variables comprised primary tumor surgery type, liver-directed treatment (resection, ablation, interventional procedures), systemic therapy regimen and cycles, targeted therapy, immunotherapy, radiotherapy, and treatment sequence (upfront surgery vs neoadjuvant systemic therapy).

Molecular variables were additionally collected when available from pathology and molecular diagnostic reports, including rat sarcoma viral oncogene homolog (RAS) mutation status, B-Raf proto-oncogene, serine/threonine kinase (BRAF) mutation status, microsatellite instability/mismatch repair (MSI/MMR) status, and human epidermal growth factor receptor 2 (HER2) amplification status. RAS status referred to Kirsten rat sarcoma viral oncogene homolog (KRAS) and/or neuroblastoma RAS viral oncogene homolog (NRAS) mutation results as reported in the clinical molecular testing record. BRAF mutation status was recorded according to available molecular reports. MSI/MMR status was determined based on immunohistochemistry for MMR proteins and/or MSI testing, as documented in the pathology or molecular diagnostic report. HER2 amplification status was recorded according to available HER2 testing results, including immunohistochemistry and/or *in situ* hybridization when performed. Because molecular testing was not uniformly performed during the study period and was partly guided by clinical indications, these variables were considered supplementary molecular characteristics and were analyzed descriptively and exploratorily rather than incorporated into the primary multivariable model.

### 2.5 Follow-up and endpoints

Follow-up commenced on the date of colon cancer diagnosis, defined as the date of histopathological confirmation of the primary tumor (from endoscopic biopsy or surgical specimen). Patients were followed until the earliest of the following events: diagnosis of LM, death from any cause, the date of last available follow-up, or December 31, 2025. Follow-up information was ascertained through outpatient clinic visits, structured telephone interviews, and review of interval imaging examinations documented in the medical record and radiology system. Imaging-based surveillance (contrast-enhanced CT and/or MRI) and clinically indicated reassessments were used to support endpoint determination. The primary endpoint for the present analyses was the occurrence of LM during the observation period; overall survival and other time-to-event outcomes were recorded when available and were analyzed according to prespecified definitions.

### 2.6 Statistical analysis

All statistical analyses were performed using IBM SPSS Statistics, version 26.0 (IBM Corp., Armonk, NY, USA). Continuous variables were assessed for distributional characteristics and are presented as mean ± standard deviation or median with interquartile range, as appropriate. Categorical variables are presented as counts and percentages. A pragmatic 1:2 non-propensity-score individual matching procedure was used to construct the matched cohort. Patients with LM were matched to non-LM controls without replacement using a nearest-neighbor strategy based on prespecified baseline variables, including age at diagnosis, sex, year of diagnosis, primary tumor location, and adequacy of follow-up for excluding LM. Tumor stage, pathological risk factors, tumor markers, inflammatory indices, and treatment variables were not used for matching because they represented candidate predictors, disease-burden indicators, or downstream management factors. Covariate balance before and after matching was evaluated using standardized mean differences (SMDs), with absolute SMD <0.10 indicating good balance and 0.10–0.20 indicating minor residual imbalance. Variables with clinically relevant residual imbalance were further considered in multivariable regression adjustment. Between-group comparisons in the matched cohort were performed using the independent-samples t test or Mann–Whitney U test for continuous variables and the Pearson χ² test or Fisher’s exact test for categorical variables. Univariable logistic regression was used to identify factors associated with LM. Variables with P<0.10 in univariable analysis and clinically relevant covariates were entered into the multivariable logistic regression model. Adjusted odds ratios and 95% confidence intervals were reported. CEA and CA19–9 were log2-transformed and interpreted per doubling. Multicollinearity was assessed using variance inflation factors. Sensitivity and subgroup analyses were conducted to evaluate the robustness of the primary findings, and available molecular data were analyzed descriptively and exploratorily because of incomplete testing. All tests were two-sided, with P<0.05 considered statistically significant.

## 3 Results

### 3.1 Study flow and cohort characteristics

A total of 814 consecutive patients with a clinical suspicion of colon cancer were screened for eligibility. After verification of diagnostic records, pathology reports, baseline staging documentation, and follow-up availability, 58 patients were excluded according to prespecified criteria. The reasons for exclusion were as follows: non–colon primary malignancy (including rectal or appendiceal tumors and other non-colonic primaries; n=19), insufficient baseline or outcome-defining data that precluded variable definition and endpoint ascertainment (including substantial missing clinicopathologic or laboratory data; n=21), and lack of evaluable hepatic imaging for metastatic assessment at baseline and/or during follow-up (no contrast-enhanced CT/MRI or images deemed non-evaluable; n=18). Consequently, 756 patients with pathologically confirmed colon cancer were included in the final analytic cohort.

During follow-up through December 31, 2025, 101 patients developed LM, yielding an overall LM incidence of 13.4% (101/756). The remaining patients without LM served as the sampling frame for control selection. A pragmatic 1:2 matching strategy, rather than propensity score matching, was applied, resulting in a final matched analysis set of 303 patients, including 101 LM cases and 202 non-LM controls. Overall follow-up time was calculated from the date of colon cancer diagnosis to LM detection, death, last contact, or administrative censoring on December 31, 2025, with a median follow-up of 29 months (IQR, 18–41 months) and a range of 3–72 months.

Before matching, several demographic and clinical variables showed imbalance between LM patients and the full non-LM sampling frame, with the largest imbalances observed for smoking status, age, diagnosis year, and sex. After pragmatic 1:2 matching, baseline comparability improved substantially, and all assessed matching variables had absolute standardized mean differences below 0.10, indicating good post-matching balance. Tumor-stage variables, adverse pathological features, tumor markers, inflammatory indices, and treatment variables were not used for matching because they were considered candidate explanatory variables, disease-burden indicators, or downstream management factors. Balance diagnostics are shown in **Supplementary Table 1**.

### 3.2 Baseline characteristics and treatment patterns

In the 1:2 matched cohort, demographic and general clinical characteristics were generally comparable between groups, including age, sex, body mass index, weight, smoking status, alcohol use, diabetes mellitus, and hypertension (all P>0.05). The proportions undergoing primary tumor surgery and the distribution of American Society of Anesthesiologists physical status among surgical patients were also similar (all P>0.05). Treatment-related variables were summarized descriptively because they may reflect disease status and downstream clinical management rather than baseline risk factors. Targeted therapy was more frequently used in the LM group than in controls (28.7% vs 9.9%, χ²=17.58, P<0.001). Among patients receiving chemotherapy, the LM group had more chemotherapy cycles (7.81 ± 2.87 vs 6.78 ± 2.65, t=2.72, P = 0.007). Treatment sequence also differed between groups, with a lower proportion of upfront surgery followed by adjuvant therapy and a higher proportion of systemic-only management in the LM group (χ²=7.46, P = 0.024) (**Table 1**).

**Table 1:** Baseline demographic, clinical, and treatment characteristics in the matched cohort.

Variable	LM (n=101)	Control (n=202)	Test statistic	P value
Age, years	60.72 ± 10.77	62.66 ± 9.32	t=-1.54	0.124
Body mass index, kg/m²	24.36 ± 3.40	24.07 ± 3.29	t=0.71	0.480
Weight, kg	69.73 ± 11.21	69.90 ± 11.69	t=-0.12	0.902
Male sex, n (%)	65 (64.4)	120 (59.4)	χ²=0.69	0.405
Smoking status, n (%)	Current 37 (36.6); Former 20 (19.8); Never 44 (43.6)	Current 57 (28.2); Former 35 (17.3); Never 110 (54.5)	χ²=3.34	0.189
Alcohol use, n (%)	Current 16 (15.8); Former 15 (14.9); Never 70 (69.3)	Current 34 (16.8); Former 23 (11.4); Never 145 (71.8)	χ²=0.74	0.690
Diabetes mellitus, n (%)	18 (17.8)	35 (17.3)	χ²=0.01	0.915
Hypertension, n (%)	38 (37.6)	78 (38.6)	χ²=0.03	0.867
Primary tumor surgery, n (%)	78 (77.2)	169 (83.7)	χ²=1.85	0.174
ASA physical status (surgery patients), n (%)	I 8 (10.3); II 53 (67.9); III 17 (21.8)	I 21 (12.4); II 109 (64.5); III 39 (23.1)	χ²=0.35	0.840
Surgical approach (surgery patients), n (%)	Laparoscopic 40 (51.3); Open 38 (48.7)	Laparoscopic 103 (60.9); Open 66 (39.1)	χ²=2.04	0.153
Chemotherapy, n (%)	85 (84.2)	151 (74.8)	χ²=3.46	0.063
Targeted therapy, n (%)	29 (28.7)	20 (9.9)	χ²=17.58	<0.001
Immunotherapy, n (%)	9 (8.9)	11 (5.4)	χ²=1.31	0.252
Radiotherapy, n (%)	8 (7.9)	11 (5.4)	χ²=0.70	0.402
Chemotherapy cycles (among treated)	7.81 ± 2.87	6.78 ± 2.65	t=2.72	0.007
Treatment sequence, n (%)	Upfront surgery→adjuvant 47 (46.5); Neoadjuvant→surgery 31 (30.7); Systemic only 23 (22.8)	Upfront surgery→adjuvant 121 (59.9); Neoadjuvant→surgery 57 (28.2); Systemic only 24 (11.9)	χ²=7.46	0.024

LM, liver metastases; ASA, American Society of Anesthesiologists.

### 3.3 Tumor burden and baseline laboratory profile according to liver metastasis status

Tumor-related characteristics differed between groups. The LM group had a larger tumor size (4.65 ± 1.67 vs 4.13 ± 1.56 cm, P = 0.010) and a more advanced local–regional disease profile, including higher T stage (P = 0.038), higher N stage (P = 0.006), and a distinct TNM stage distribution driven by stage IV classification in the LM group (P<0.001). Adverse pathological features were more frequent among LM patients, including LVI, PNI, and tumor deposits (all P<0.05). Among surgical patients, positive lymph node burden and lymph node ratio were higher in the LM group (P = 0.049 and P = 0.035, respectively), whereas the number of examined lymph nodes and resection margin status did not differ significantly. Baseline laboratory testing showed higher CEA and CA19–9 levels in the LM group (both P<0.001), accompanied by higher ALT, AST, ALP, GGT, and total bilirubin levels (all P ≤ 0.046). Inflammatory indices were also higher in LM patients, with elevated NLR and PLR (both P<0.001). These laboratory differences should be interpreted as baseline profiles associated with LM status and tumor burden, rather than as definitive predictors of future LM development, particularly because synchronous LM was included in the LM group (**Table 2**).

**Table 2:** Tumor-related pathology and baseline laboratory indices in the matched cohort.

Variable	LM (n=101)	Control (n=202)	Test statistic	P value
Tumor size, cm	4.65 ± 1.67	4.13 ± 1.56	t=2.61	0.010
Primary tumor location, n (%)	Right 38 (37.6); Transverse 14 (13.9); Left 49 (48.5)	Right 77 (38.1); Transverse 24 (11.9); Left 101 (50.0)	χ²=0.24	0.885
Histology, n (%)	Adenocarcinoma 89 (88.1); Mucinous 12 (11.9)	Adenocarcinoma 171 (84.7); Mucinous 31 (15.3)	χ²=0.66	0.415
Differentiation, n (%)	Well 14 (13.9); Moderate 56 (55.4); Poor 31 (30.7)	Well 33 (16.3); Moderate 124 (61.4); Poor 45 (22.3)	χ²=2.57	0.277
T stage, n (%)	T1 5 (5.0); T2 18 (17.8); T3 50 (49.5); T4 28 (27.7)	T1 14 (6.9); T2 58 (28.7); T3 98 (48.5); T4 32 (15.8)	χ²=8.42	0.038
N stage, n (%)	N0 21 (20.8); N1 39 (38.6); N2 41 (40.6)	N0 77 (38.1); N1 69 (34.2); N2 56 (27.7)	χ²=10.11	0.006
TNM stage, n (%)	I 1 (1.0); II 12 (11.9); III 26 (25.7); IV 62 (61.4)	I 15 (7.4); II 68 (33.7); III 119 (58.9); IV 0 (0.0)	χ²=156.86	<0.001
Lymphovascular invasion, n (%)	38 (37.6)	50 (24.8)	χ²=5.40	0.020
Perineural invasion, n (%)	39 (38.6)	54 (26.7)	χ²=4.59	0.032
Tumor deposits, n (%)	33 (32.7)	39 (19.3)	χ²=6.52	0.011
Resection margin status (surgery patients), n (%)	R0 68 (87.2); R1 6 (7.7); R2 4 (5.1)	R0 160 (94.7); R1 7 (4.1); R2 2 (1.2)	χ²=5.02	0.081
Examined lymph nodes (surgery patients)	18.01 ± 7.00	19.36 ± 7.41	t=-1.38	0.169
Positive lymph nodes (surgery patients)	3.69 ± 4.03	2.66 ± 3.17	t=1.99	0.049
Lymph node ratio (surgery patients)	0.19 ± 0.18	0.14 ± 0.15	t=2.13	0.035
CEA, ng/mL	12.61 [6.01, 26.92]	4.35 [2.07, 9.24]	Mann–Whitney U	<0.001
CA19-9, U/mL	36.63 [17.38, 82.31]	16.29 [8.26, 33.76]	Mann–Whitney U	<0.001
ALT, U/L	28.12 [17.68, 46.33]	23.74 [16.08, 37.83]	Mann–Whitney U	0.046
AST, U/L	31.08 [20.17, 52.31]	26.52 [18.25, 41.32]	Mann–Whitney U	0.030
ALP, U/L	129.98 [97.39, 181.94]	92.58 [74.63, 117.45]	Mann–Whitney U	<0.001
GGT, U/L	61.13 [37.69, 112.53]	33.80 [21.89, 56.41]	Mann–Whitney U	<0.001
Total bilirubin, μmol/L	14.02 [10.64, 19.20]	12.29 [9.44, 15.71]	Mann–Whitney U	0.003
NLR	3.59 [2.59, 5.24]	2.67 [1.99, 3.83]	Mann–Whitney U	<0.001
PLR	221.08 [167.86, 310.51]	170.59 [129.30, 241.12]	Mann–Whitney U	<0.001

TNM, tumor–node–metastasis; CEA, carcinoembryonic antigen; CA19-9, carbohydrate antigen 19-9; ALT, alanine aminotransferase; AST, aspartate aminotransferase; ALP, alkaline phosphatase; GGT, gamma-glutamyl transferase; NLR, neutrophil-to-lymphocyte ratio; PLR, platelet-to-lymphocyte ratio.

### 3.4 Univariable analysis: candidate factors associated with liver metastasis

In univariable logistic regression models, several variables showed statistically significant associations with LM status. Smoking was associated with higher odds of LM (current vs never: OR 3.06; former vs never: OR 2.70). Adverse pathological features, including LVI, PNI, and tumor deposits, were also associated with higher odds of LM. Advanced local and nodal disease showed similar associations, particularly T4 stage versus T1 and N2 stage versus N0. Biomarker and inflammation-related variables showed consistent directionality: higher CEA and CA19–9 levels, each modeled per doubling, higher NLR, and higher platelet-to-lymphocyte ratio were associated with higher odds of LM status, whereas higher albumin was inversely associated with LM. Treatment variables reflecting more intensive systemic management, including targeted therapy and immunotherapy, were associated with higher odds of LM in univariable analyses; these findings were interpreted descriptively because treatment patterns may be influenced by metastatic status and disease severity. Based on the prespecified screening threshold of P<0.10, variables marked as eligible were considered for inclusion in the multivariable model, with additional retention of clinically relevant covariates as needed (**Table 3**).

**Table 3:** Univariable logistic regression for liver metastasis status.

Candidate factor	OR (95% CI)	P value	Handling of continuous variables	Enter multivariable (P<0.10)
Age (per 10-year increase)	0.73 (0.57–0.92)	0.008	Modeled as continuous (per 10 years)	Yes
Male sex (vs female)	1.00 (0.62–1.62)	1.000	Binary indicator	No
Body mass index (per 1 kg/m² increase)	1.03 (0.96–1.10)	0.439	Modeled as continuous (per 1 kg/m²)	No
Diabetes mellitus (yes vs no)	1.18 (0.64–2.17)	0.598	Binary indicator	No
Hypertension (yes vs no)	0.81 (0.49–1.33)	0.400	Binary indicator	No
Smoking: Current (vs Never)	3.06 (1.76–5.29)	<0.001	Categorical; reference=Never	Yes
Smoking: Former (vs Never)	2.70 (1.37–5.33)	0.004	Categorical; reference=Never	Yes
Alcohol: Current (vs Never)	0.75 (0.38–1.48)	0.410	Categorical; reference=Never	No
Alcohol: Former (vs Never)	1.65 (0.82–3.33)	0.159	Categorical; reference=Never	No
Tumor size (per 1 cm increase)	1.11 (0.95–1.30)	0.176	Modeled as continuous (per 1 cm)	No
Lymphovascular invasion (yes vs no)	1.75 (1.04–2.95)	0.037	Binary indicator	Yes
Perineural invasion (yes vs no)	2.17 (1.31–3.59)	0.003	Binary indicator	Yes
Tumor deposits (yes vs no)	2.32 (1.33–4.02)	0.003	Binary indicator	Yes
T stage: T2 (vs T1)	1.35 (0.27–6.73)	0.711	Categorical; reference=T1	No
T stage: T3 (vs T1)	2.70 (0.58–12.63)	0.208	Categorical; reference=T1	No
T stage: T4 (vs T1)	7.56 (1.53–37.30)	0.013	Categorical; reference=T1	Yes
N stage: N1 (vs N0)	1.69 (0.93–3.10)	0.087	Categorical; reference=N0	Yes
N stage: N2 (vs N0)	2.25 (1.21–4.16)	0.010	Categorical; reference=N0	Yes
Lymph node ratio (per 0.1 increase; surgery patients)	1.09 (0.90–1.32)	0.394	Modeled as continuous; scaled per 0.1 increase (surgery patients only)	No
CEA (per doubling; log2-transformed)	1.86 (1.55–2.24)	<0.001	Log2-transformed; OR per doubling	Yes
CA19-9 (per doubling; log2-transformed)	1.60 (1.35–1.91)	<0.001	Log2-transformed; OR per doubling	Yes
Neutrophil-to-lymphocyte ratio (per 1-unit increase)	1.35 (1.18–1.55)	<0.001	Modeled as continuous (per 1 unit)	Yes
Platelet-to-lymphocyte ratio (per 50-unit increase)	1.33 (1.17–1.51)	<0.001	Scaled; OR per 50 units	Yes
Albumin (per 1 g/L increase)	0.93 (0.88–0.99)	0.021	Modeled as continuous (per 1 g/L)	Yes
Chemotherapy (yes vs no)	1.44 (0.81–2.58)	0.218	Binary indicator	No
Targeted therapy (yes vs no)	5.27 (2.68–10.37)	<0.001	Binary indicator	Yes
Immunotherapy (yes vs no)	3.76 (1.43–9.86)	0.007	Binary indicator	Yes

OR, odds ratio; CI, confidence interval; CEA, carcinoembryonic antigen; CA19-9, carbohydrate antigen 19-9; NLR, neutrophil-to-lymphocyte ratio; PLR, platelet-to-lymphocyte ratio.

### 3.5 Multivariable analysis: independent factors associated with liver metastasis

In the multivariable logistic regression model, several pathological, nodal, and biomarker-related variables were independently associated with LM status. LVI remained associated with higher odds of LM after adjustment (adjusted OR 2.57, 95% CI 1.38–4.76; P = 0.003). PNI was also associated with higher odds of LM (adjusted OR 1.94, 95% CI 1.05–3.59; P = 0.035). Compared with N0 disease, N2 stage showed an independent association with LM status (adjusted OR 2.48, 95% CI 1.17–5.25; P = 0.018), whereas N1 stage did not reach statistical significance (P = 0.161). Tumor deposits showed a borderline association (adjusted OR 1.89, 95% CI 0.97–3.67; P = 0.061). Among laboratory variables, higher CEA levels, modeled per doubling, were associated with higher odds of LM status (adjusted OR 1.72, 95% CI 1.42–2.10; P<0.001), as was higher NLR (adjusted OR 1.35 per 1-unit increase, 95% CI 1.15–1.59; P<0.001). These laboratory associations should be interpreted as markers related to LM status and tumor burden rather than definitive predictors of future LM development. Age showed an inverse adjusted association (adjusted OR 0.67 per 10-year increase, 95% CI 0.50–0.89; P = 0.007). Smoking status, T4 stage, and albumin did not show statistically significant independent associations in the adjusted model. Model diagnostics indicated no clinically meaningful multicollinearity, with a maximum variance inflation factor of approximately 1.43. Correlated laboratory indices were not simultaneously retained to reduce redundancy and improve model interpretability (**Table 4**).

**Table 4:** Multivariable logistic regression for liver metastasis status.

Predictor	Adjusted OR	95% CI	P value
Age (per 10-year increase)	0.67	0.50–0.89	0.007
Smoking: Current (vs Never)	1.08	0.55–2.13	0.824
Smoking: Former (vs Never)	1.71	0.77–3.80	0.186
Lymphovascular invasion (yes vs no)	2.57	1.38–4.76	0.003
Perineural invasion (yes vs no)	1.94	1.05–3.59	0.035
Tumor deposits (yes vs no)	1.89	0.97–3.67	0.061
T4 stage (vs T1–T3)	1.46	0.69–3.10	0.320
N stage: N1 (vs N0)	1.68	0.81–3.49	0.161
N stage: N2 (vs N0)	2.48	1.17–5.25	0.018
CEA (per doubling; log2-transformed)	1.72	1.42–2.10	<0.001
NLR (per 1-unit increase)	1.35	1.15–1.59	<0.001
Albumin (per 1 g/L increase)	0.97	0.90–1.04	0.367

OR, odds ratio; CI, confidence interval; CEA, carcinoembryonic antigen; NLR, neutrophil-to-lymphocyte ratio.

### 3.6 Subgroup analysis: synchronous versus metachronous liver metastasis

Among the 101 patients with LM, 62 (61.4%) met the criteria for synchronous LM, defined as LM identified at diagnosis or within 6 months, and 39 (38.6%) were classified as having metachronous LM, defined as LM detected more than 6 months after diagnosis during follow-up. Clinicopathological and laboratory profiles were compared between these two LM subgroups, and an exploratory multivariable logistic regression model was fitted with metachronous LM as the dependent variable.

Compared with metachronous LM, synchronous LM showed a laboratory profile compatible with greater tumor burden and systemic inflammation, including higher CEA (P = 0.017) and higher NLR (P<0.001). N stage distribution differed between subgroups (P = 0.017), with a higher proportion of N2 disease in synchronous LM. PNI showed a borderline difference (P = 0.050), whereas age, albumin, and LVI did not differ significantly between synchronous and metachronous LM (all P>0.05) (**Supplementary Table 2**).

In the exploratory adjusted model, higher CEA and higher NLR were inversely associated with metachronous versus synchronous LM (adjusted OR 0.72, P = 0.033; and adjusted OR 0.44, P<0.001, respectively), suggesting that higher baseline tumor marker levels and inflammatory activity were more characteristic of synchronous presentation in this cohort. Age and LVI showed borderline associations (P = 0.077 and P = 0.092). For N stage, the direction of association suggested lower odds of metachronous LM among patients with N2 disease compared with N0 disease (P = 0.056), although this did not reach conventional statistical significance. Collinearity diagnostics showed low variance inflation factors (VIF range, 1.03–2.16). These subgroup findings should be interpreted as exploratory associations rather than causal or predictive evidence (**Table 5**).

**Table 5:** Multivariable logistic regression for metachronous liver metastasis (reference: synchronous liver metastasis).

Predictor	Adjusted OR	95% CI	P value
CEA (per doubling; log2-transformed)	0.72	0.54–0.97	0.033
NLR (per 1-unit increase)	0.44	0.28–0.69	<0.001
Age (per 1-year increase)	1.04	1.00–1.08	0.077
Lymphovascular invasion (yes vs no)	0.41	0.14–1.16	0.092
N stage: N1 (vs N0)	0.36	0.09–1.45	0.151
N stage: N2 (vs N0)	0.24	0.05–1.04	0.056

CEA, carcinoembryonic antigen; NLR, neutrophil-to-lymphocyte ratio; OR, odds ratio; CI, confidence interval; VIF, variance inflation factor.

### 3.7 Sensitivity analyses

Sensitivity and supplementary analyses yielded results generally consistent with the primary multivariable model. In the complete-case analysis and the multiple-imputation pooled analysis, the direction and magnitude of associations were comparable to those of the primary model. CEA, NLR, LVI, PNI, and N2 stage showed similar patterns of association with LM status across these analyses (**Supplementary Table 3**). Restriction to patients with pathologic staging after surgery produced similar estimates for the key variables, suggesting that the findings were unlikely to be explained solely by clinical-staging misclassification. Follow-up-based restrictions among controls, including exclusion of short follow-up and requiring a minimum follow-up duration, also yielded similar estimates (**Supplementary Table 4**). Alternative specifications of continuous predictors, including quartile categorization and log-transformed models, showed consistent associations for CEA and NLR (**Supplementary Table 5**). Overall, these analyses supported the robustness of the observed associations across different missing-data strategies, staging subsets, follow-up restrictions, and functional-form assumptions.

### 3.8 Supplementary molecular characterization

Molecular data were available for a subset of patients and were therefore analyzed descriptively and in an exploratory manner (**Supplementary Tables 6**, **S7**). In the matched cohort, RAS mutation testing was available in 195 patients, BRAF mutation testing in 178 patients, MSI/MMR status in 211 patients, and HER2 amplification status in 99 patients. The proportion of patients with available molecular testing was higher in the LM group than in controls, likely reflecting clinically driven testing patterns in patients with metastatic disease.

Among tested patients, RAS mutations were observed in 39 of 77 LM patients and 50 of 118 controls (50.6% vs 42.4%, P = 0.257). BRAF mutations were present in 8 of 72 LM patients and 9 of 106 controls (11.1% vs 8.5%, P = 0.559). MSI-H/dMMR status was numerically less frequent in the LM group than in controls, but the difference was not statistically significant (6.3% vs 12.9%, P = 0.132). HER2 amplification was uncommon in both groups (4.4% vs 3.7%, P = 0.852). Right-sided tumor location was similarly distributed between groups (37.6% vs 38.1%, P = 0.933).

In the exploratory extended model based on patients with available molecular data, the clinicopathologic and laboratory associations were directionally consistent with those observed in the primary model. CEA and NLR remained associated with LM, whereas RAS mutation, BRAF mutation, MSI-H/dMMR status, and right-sided tumor location showed no statistically significant independent association with LM in this subset. These findings indicate that the available molecular data were broadly consistent with the primary clinicopathologic and laboratory findings. However, because molecular testing was incomplete, clinically selected, and not uniformly available across the cohort, these exploratory results should be interpreted cautiously and regarded as hypothesis-generating.

## 4 Discussion

In this retrospective matched cohort study of patients with colon cancer, we characterized the clinical, pathological, and laboratory profile associated with LM status using routinely available diagnostic variables. The main contribution of this study is the integrated assessment of tumor invasiveness, nodal burden, serum tumor markers, and systemic inflammatory indices within a pragmatically matched cohort, with additional distinction between synchronous and metachronous LM. This approach provides a clinically oriented framework for interpreting LM-related profiles at the time of diagnosis and follow-up, while avoiding overreliance on any single variable. Because the identified variables are commonly available in routine practice, these findings may help clinicians recognize patients who warrant more careful hepatic assessment, individualized surveillance planning, and multidisciplinary evaluation. Importantly, the observed associations should be interpreted as markers of LM status and disease burden rather than definitive predictors of future metastasis (1, 18).

The present results suggest that LM in colon cancer is associated with a convergent clinicopathological and laboratory phenotype rather than a single isolated feature. After pragmatic matching, baseline demographic and general clinical characteristics were broadly comparable between LM and non-LM patients, supporting a more focused interpretation of tumor- and biomarker-related differences. LM patients showed larger tumors, more advanced T and N stage distributions, higher positive lymph node burden, and more frequent LVI, PNI, and tumor deposits. In the adjusted model, LVI, PNI, and N2 stage remained independently associated with LM status, indicating that local invasion, perineural spread, and nodal disease burden were closely linked to hepatic metastatic presentation. These findings are biologically coherent, as these pathological features reflect aggressive tumor behavior and potential access to lymphovascular or perineural routes of dissemination. Importantly, T4 stage and smoking were significant in univariable analysis but not retained as independent associations after adjustment, suggesting that their apparent relationships may be partly explained by coexisting pathological or biomarker features. The inverse association between age and LM status should be interpreted cautiously as an adjusted statistical association, potentially reflecting differences in diagnostic intensity, tumor biology, treatment pathways, or competing clinical factors rather than a direct protective effect (19, 20). Overall, the main model supports the clinical value of integrating adverse pathological features with nodal stage when evaluating the likelihood of hepatic involvement in colon cancer patients (21, 22).

The laboratory findings further indicate that LM status was accompanied by a distinct baseline biomarker profile. CEA remained strongly associated with LM after adjustment and showed consistent results across complete-case, multiple-imputation, surgical-only, follow-up-restricted, and alternative functional-form analyses. This consistency suggests that CEA is a robust marker of metastatic disease burden in this cohort when interpreted as an associated baseline indicator rather than a definitive predictor of future LM (23). NLR also remained independently associated with LM status and showed graded associations in quartile-based analyses, supporting the relevance of systemic inflammatory status in the clinical profile of patients with hepatic metastatic involvement (24, 25). The subgroup analysis provided additional interpretive value by distinguishing synchronous from metachronous LM. Synchronous LM was characterized by higher CEA, higher NLR, and a greater proportion of N2 disease, suggesting a presentation more compatible with established tumor burden and systemic inflammation at or near diagnosis. By contrast, metachronous LM appeared less strongly linked to these elevated baseline biomarker patterns, which may reflect different timing of metastatic detection or a less overt disease-burden profile at initial assessment. Treatment-related differences, including more frequent targeted therapy and systemic-only management in the LM group, are best understood as descriptive reflections of metastatic status and clinical decision-making rather than baseline risk factors (26, 27). Finally, the exploratory molecular analysis showed that the main clinicopathological and laboratory associations were directionally preserved in the molecularly characterized subset, whereas RAS, BRAF, MSI/MMR status, and right-sided tumor location were not independently associated with LM in that subset. This supports the practical relevance of routinely available pathological and laboratory variables for baseline assessment and surveillance planning (28, 29).

This study has several methodological and clinical strengths. The use of a consecutively enrolled colon cancer cohort, predefined outcome definitions, imaging-based LM ascertainment, and source-document verification enhanced the reliability of data extraction and endpoint classification. In addition, pragmatic 1:2 matching with standardized balance diagnostics improved baseline comparability between LM and non-LM patients, while multiple sensitivity analyses supported the consistency of the main associations across different analytic assumptions. Clinically, the study emphasizes the value of routinely available information obtained during standard diagnostic work-up, including pathological assessment, nodal staging, serum tumor markers, and inflammatory indices. These variables do not require specialized testing and may therefore provide a practical framework for identifying patients who warrant closer hepatic evaluation, individualized follow-up intensity, and early multidisciplinary discussion. Given the retrospective observational design, these findings should be used as supportive clinical information rather than as evidence of causality or as a standalone predictive tool. Beyond the routinely available clinicopathological and laboratory variables evaluated in the present study, contemporary assessment of colorectal LM increasingly incorporates imaging-derived biomarkers. Recent studies suggest that radiomics features extracted from contrast-enhanced magnetic resonance imaging, including hepatobiliary phase imaging, may provide complementary information on tumor heterogeneity, biological behavior, recurrence risk, mutational status, and pathological characteristics in patients with colorectal LM (30, 31). Such non-invasive approaches may refine risk stratification and treatment selection by capturing imaging phenotypes that are not fully represented by conventional clinical or laboratory parameters. Therefore, future studies integrating clinicopathological factors, serum biomarkers, molecular profiles, and quantitative imaging features may provide a more comprehensive framework for individualized assessment of liver metastasis in colon cancer.

Several limitations should be considered. First, the retrospective observational design limited causal inference and temporal interpretation, and residual confounding may remain despite pragmatic matching and balance assessment. Because both synchronous and metachronous LM were included, laboratory variables such as liver enzymes, bilirubin, CEA, carbohydrate antigen 19-9, and inflammation-based indices may reflect established or occult metastatic disease and disease burden, particularly in synchronous LM, rather than true predictors of future metastasis. Second, outcome ascertainment may have been influenced by heterogeneity in surveillance intensity, as patients with more aggressive disease, abnormal biomarkers, or clinical suspicion of metastasis may have undergone more frequent imaging. Although controls were required to have evaluable hepatic imaging or adequate follow-up, and follow-up-restricted sensitivity analyses showed consistent findings, residual detection bias cannot be excluded. Third, treatment-related variables likely reflected confounding by indication, metastatic status, and downstream clinical decision-making, and should not be interpreted as etiologic determinants of LM. Fourth, molecular characterization was incomplete and clinically selected, and detailed radiologic measures of liver tumor burden were unavailable, limiting a more comprehensive evaluation of molecular and imaging-related metastatic phenotypes. Finally, this study identified variables associated with LM status but did not develop or validate a clinically applicable prediction model; therefore, discrimination, calibration, and decision-curve analyses were not performed. Future prospective, multicenter studies with standardized imaging surveillance, harmonized pathology assessment, complete molecular profiling, detailed radiologic tumor-burden evaluation, and integration of pathological, laboratory, imaging-derived, and molecular variables are needed to develop and externally validate clinically applicable risk models for LM assessment in colon cancer.

## 5 Conclusion

In this matched cohort of patients with colon cancer, liver metastasis status was independently associated with invasion-related pathological features, including LVI and PNI, high nodal burden represented by N2 stage, and routinely available biomarkers compatible with tumor burden and systemic inflammation, including CEA and NLR. Synchronous liver metastasis showed higher CEA and NLR levels and a higher prevalence of N2 disease than metachronous liver metastasis, suggesting potential heterogeneity in clinical profiles according to the timing of metastatic presentation. The consistency of these associations across multiple sensitivity analyses further supports the robustness of the observed findings. These results may help inform clinical assessment, baseline hepatic evaluation, individualized surveillance planning, and multidisciplinary management in patients with colon cancer.

## Data Availability

The raw data supporting the conclusions of this article will be made available by the authors, without undue reservation.
