# Supplementary material for: Clinical characteristics and risk factors in patients with colon cancer liver metastases
Source: Front Oncol. 2026 Jul 22;16:1817217. doi: 10.3389/fonc.2026.1817217 (PMC13437466; doi:10.3389/fonc.2026.1817217)
Supplement: Supplementary file 1 [file Table1.docx]

Supplementary Table S1. Balance diagnostics before and after pragmatic 1:2 matching

| Variable | Before matching: LM (n=101) | Before matching: non-LM pool (n=655) | SMD before matching | After matching: LM (n=101) | After matching: control (n=202) | SMD after matching |
| --- | --- | --- | --- | --- | --- | --- |
| Age, years | 60.72 ± 10.77 | 64.10 ± 9.80 | 0.328 | 60.72 ± 10.77 | 61.43 ± 9.87 | 0.069 |
| Male sex, n (%) | 65 (64.4) | 352 (53.7) | 0.217 | 65 (64.4) | 126 (62.4) | 0.041 |
| Body mass index, kg/m² | 24.36 ± 3.40 | 23.85 ± 3.24 | 0.154 | 24.36 ± 3.40 | 24.21 ± 3.31 | 0.045 |
| Weight, kg | 69.73 ± 11.21 | 68.40 ± 11.40 | 0.118 | 69.73 ± 11.21 | 69.40 ± 11.56 | 0.029 |
| Diagnosis year, n (%) | 2020: 20 (19.8); 2021: 23 (22.8); 2022: 27 (26.7); 2023: 31 (30.7) | 2020: 203 (31.0); 2021: 170 (26.0); 2022: 151 (23.1); 2023: 131 (20.0) | 0.259 | 2020: 20 (19.8); 2021: 23 (22.8); 2022: 27 (26.7); 2023: 31 (30.7) | 2020: 42 (20.8); 2021: 46 (22.8); 2022: 54 (26.7); 2023: 60 (29.7) | 0.024 |
| Primary tumor location, n (%) | Right: 38 (37.6); Transverse: 14 (13.9); Left: 49 (48.5) | Right: 294 (44.9); Transverse: 62 (9.5); Left: 299 (45.6) | 0.148 | Right: 38 (37.6); Transverse: 14 (13.9); Left: 49 (48.5) | Right: 77 (38.1); Transverse: 26 (12.9); Left: 99 (49.0) | 0.030 |
| Smoking status, n (%) | Current: 37 (36.6); Former: 20 (19.8); Never: 44 (43.6) | Current: 147 (22.4); Former: 104 (15.9); Never: 404 (61.7) | 0.369 | Current: 37 (36.6); Former: 20 (19.8); Never: 44 (43.6) | Current: 72 (35.6); Former: 40 (19.8); Never: 90 (44.6) | 0.022 |
| Alcohol use, n (%) | Current: 16 (15.8); Former: 15 (14.9); Never: 70 (69.3) | Current: 93 (14.2); Former: 68 (10.4); Never: 494 (75.4) | 0.137 | Current: 16 (15.8); Former: 15 (14.9); Never: 70 (69.3) | Current: 31 (15.3); Former: 31 (15.3); Never: 140 (69.3) | 0.012 |
| Diabetes mellitus, n (%) | 18 (17.8) | 146 (22.3) | 0.112 | 18 (17.8) | 36 (17.8) | 0.000 |
| Hypertension, n (%) | 38 (37.6) | 301 (46.0) | 0.170 | 38 (37.6) | 76 (37.6) | 0.000 |

Abbreviations: LM, liver metastases; non-LM, non-liver metastases; SMD, standardized mean difference; BMI, body mass index; n, number.

Supplementary Table S2. Comparison between synchronous and metachronous LM (LM patients only; n=101).

| Variable | Synchronous (n=62) | Metachronous (n=39) | Test statistic | P value |
| --- | --- | --- | --- | --- |
| Age, years | 57.78 ± 12.33 | 61.07 ± 12.21 | t=-1.31 | 0.193 |
| CEA, ng/mL | 19.26 [7.67, 33.48] | 9.97 [3.96, 15.93] | U=1553 | 0.017 |
| NLR | 4.03 [3.43, 5.18] | 2.89 [2.52, 3.53] | U=1835 | <0.001 |
| Albumin, g/L | 38.03 ± 4.33 | 38.93 ± 4.37 | t=-1.02 | 0.312 |
| Lymphovascular invasion, n (%) | 24 (38.7) | 10 (25.6) | χ²=1.29 | 0.256 |
| Perineural invasion, n (%) | 31 (50.0) | 11 (28.2) | χ²=3.83 | 0.050 |
| N stage, n (%) | N0 6 (9.7); N1 26 (41.9); N2 30 (48.4) | N0 11 (28.2); N1 18 (46.2); N2 10 (25.6) | χ²=8.11 | 0.017 |

CEA, carcinoembryonic antigen; NLR, neutrophil-to-lymphocyte ratio

Supplementary Table S3. Consistency of multivariable results across missing-data strategies and analytic subsets.

| Predictor | Primary model aOR | 95% CI | P value | Complete-case aOR | 95% CI | P value | MI-pooled aOR | 95% CI | P value |
| --- | --- | --- | --- | --- | --- | --- | --- | --- | --- |
| Age (per 10-year increase) | 0.67 | 0.50–0.89 | 0.007 | 0.69 | 0.50–0.95 | 0.023 | 0.68 | 0.52–0.90 | 0.008 |
| Lymphovascular invasion (yes vs no) | 2.57 | 1.38–4.76 | 0.003 | 2.41 | 1.22–4.75 | 0.011 | 2.49 | 1.35–4.61 | 0.004 |
| Perineural invasion (yes vs no) | 1.94 | 1.05–3.59 | 0.035 | 1.88 | 0.97–3.66 | 0.061 | 1.92 | 1.05–3.50 | 0.033 |
| N stage: N2 (vs N0) | 2.48 | 1.17–5.25 | 0.018 | 2.36 | 1.04–5.36 | 0.040 | 2.44 | 1.17–5.08 | 0.017 |
| CEA (per doubling; log2-transformed) | 1.72 | 1.42–2.10 | <0.001 | 1.66 | 1.34–2.06 | <0.001 | 1.70 | 1.41–2.05 | <0.001 |
| NLR (per 1-unit increase) | 1.35 | 1.15–1.59 | <0.001 | 1.32 | 1.11–1.57 | 0.002 | 1.34 | 1.15–1.56 | <0.001 |
| Albumin (per 1 g/L increase) | 0.97 | 0.90–1.04 | 0.367 | 0.96 | 0.89–1.04 | 0.318 | 0.97 | 0.91–1.04 | 0.409 |

CEA, carcinoembryonic antigen; NLR, neutrophil-to-lymphocyte ratio

Supplementary Table S4. Restriction to pathologically staged surgical patients and follow-up robustness.

| Analysis | N (events) | Key predictors retained in model | CEA (per doubling) aOR (95% CI), P | NLR (per 1-unit) aOR (95% CI), P | LVI aOR (95% CI), P | N2 (vs N0) aOR (95% CI), P |
| --- | --- | --- | --- | --- | --- | --- |
| Surgical-only (pathologic T/N available) | 243 (78) | Age, LVI, PNI, N stage, CEA, NLR | 1.68 (1.33–2.12), <0.001 | 1.29 (1.06–1.58), 0.010 | 2.35 (1.18–4.70), 0.015 | 2.21 (1.00–4.87), 0.049 |
| Excluding short follow-up controls* | 276 (101) | Age, LVI, PNI, N stage, CEA, NLR | 1.74 (1.44–2.12), <0.001 | 1.36 (1.16–1.60), <0.001 | 2.52 (1.36–4.65), 0.003 | 2.46 (1.15–5.26), 0.021 |
| Minimum follow-up ≥12 months in controls* | 261 (101) | Age, LVI, PNI, N stage, CEA, NLR | 1.71 (1.41–2.08), <0.001 | 1.33 (1.13–1.57), 0.001 | 2.44 (1.31–4.52), 0.005 | 2.39 (1.11–5.12), 0.026 |

CEA, carcinoembryonic antigen; NLR, neutrophil-to-lymphocyte ratio; LVI, lymphovascular invasion; PNI, perineural invasion.

*For these follow-up sensitivity analyses, LM cases were retained; restrictions were applied to controls to reduce outcome misclassification due to limited observation time.

Supplementary Table S5. Alternative handling of continuous variables (quantile categorization vs transformation).

| Predictor specification | Reference | Comparison | Adjusted OR | 95% CI | P value |
| --- | --- | --- | --- | --- | --- |
| CEA (quartiles) | Q1 (lowest) | Q2 | 1.42 | 0.72–2.81 | 0.312 |
|  | Q1 (lowest) | Q3 | 2.64 | 1.34–5.20 | 0.005 |
|  | Q1 (lowest) | Q4 (highest) | 5.48 | 2.72–11.05 | <0.001 |
| NLR (quartiles) | Q1 (lowest) | Q2 | 1.58 | 0.79–3.17 | 0.194 |
|  | Q1 (lowest) | Q3 | 2.71 | 1.36–5.42 | 0.005 |
|  | Q1 (lowest) | Q4 (highest) | 4.96 | 2.38–10.32 | <0.001 |
| CEA (log-transformed, continuous) | — | per 1-unit increase in ln(CEA) | 1.62 | 1.32–1.98 | <0.001 |
| NLR (log-transformed, continuous) | — | per 1-unit increase in ln(NLR) | 1.89 | 1.33–2.69 | <0.001 |

aOR, adjusted odds ratio; CI, confidence interval; MI, multiple imputation; CEA, carcinoembryonic antigen; NLR, neutrophil-to-lymphocyte ratio

Supplementary Table S6. Molecular characteristics and tumor sidedness in the matched cohort

| Variable | LM group | Control group | Available n | Missing / not tested, n (%) | P value |
| --- | --- | --- | --- | --- | --- |
| RAS mutation | 39/77 (50.6) | 50/118 (42.4) | 195 | 108/303 (35.6) | 0.257 |
| BRAF mutation | 8/72 (11.1) | 9/106 (8.5) | 178 | 125/303 (41.3) | 0.559 |
| MSI-H/dMMR | 5/79 (6.3) | 17/132 (12.9) | 211 | 92/303 (30.4) | 0.132 |
| HER2 amplification | 2/45 (4.4) | 2/54 (3.7) | 99 | 204/303 (67.3) | 0.852 |
| Right-sided tumor | 38/101 (37.6) | 77/202 (38.1) | 303 | 0/303 (0.0) | 0.933 |

Abbreviations: LM, liver metastases; RAS, KRAS/NRAS mutation status; BRAF, B-Raf proto-oncogene, serine/threonine kinase; MSI-H, microsatellite instability-high; dMMR, deficient mismatch repair; HER2, human epidermal growth factor receptor 2.

Supplementary Table S7. Exploratory extended logistic regression model in the molecularly characterized subset

| Predictor | Adjusted OR | 95% CI | P value |
| --- | --- | --- | --- |
| Age, per 10-year increase | 0.70 | 0.49–1.01 | 0.056 |
| Lymphovascular invasion, yes vs no | 2.35 | 1.08–5.12 | 0.032 |
| Perineural invasion, yes vs no | 1.74 | 0.81–3.75 | 0.154 |
| N2 stage, vs N0 | 2.27 | 0.95–5.46 | 0.066 |
| CEA, per doubling | 1.63 | 1.25–2.14 | <0.001 |
| NLR, per 1-unit increase | 1.29 | 1.04–1.60 | 0.020 |
| RAS mutation, yes vs no | 1.33 | 0.65–2.72 | 0.435 |
| BRAF mutation, yes vs no | 1.18 | 0.38–3.72 | 0.776 |
| MSI-H/dMMR, yes vs no | 0.54 | 0.16–1.82 | 0.321 |
| Right-sided tumor, yes vs no | 0.96 | 0.46–2.01 | 0.913 |

Abbreviations: OR, odds ratio; CI, confidence interval; CEA, carcinoembryonic antigen; NLR, neutrophil-to-lymphocyte ratio; RAS, KRAS/NRAS mutation status; BRAF, B-Raf proto-oncogene, serine/threonine kinase; MSI-H, microsatellite instability-high; dMMR, deficient mismatch repair; N0/N2, regional lymph node categories according to the tumor–node–metastasis classification.
